# Response of Spring Wheat (*Triticum aestivum* L.) Quality Traits and Yield to Sowing Date

**DOI:** 10.1371/journal.pone.0126097

**Published:** 2015-04-30

**Authors:** Mukhtar Ahmed, Fayyaz-ul-Hassan

**Affiliations:** 1 Department of Agronomy, Pir Mehr Ali Shah Arid Agriculture University, Rawalpindi, Punjab, Pakistan; 2 Department of Biological System Engineering, Washington State University, Pullman, Washington, United States of America; 3 Department of Biosciences, COMSATS, CIIT, Park Road, Islamabad, Pakistan; Institute of Genetics and Developmental Biology, CHINA

## Abstract

The unpredictability and large fluctuation of the climatic conditions in rainfed regions do affect spring wheat yield and grain quality. These variations offer the opportunity for the production of better quality wheat. The effect of variable years, locations and sowing managements on wheat grain yield and quality was studied through field experiments using three genotypes, three locations for two years under rainfed conditions. The two studied years as contrasting years at three locations and sowing dates depicted variability in temperature and water stress during grain filling which resulted considerable change in grain yield and quality. Delayed sowing, years (2009–10) and location (Talagang) with high temperature and water stress resulted increased proline, and grain quality traits i.e. grain protein (GP) and grain ash (GA) than optimum conditions (during 2008–09, at Islamabad and early sowing). However, opposite trend was observed for dry gluten (DG), sodium dodecyl sulphate (SDS), SPAD content and grain yield irrespective of genotypes. The influence of variable climatic conditions was dominant in determining the quality traits and inverse relationship was observed among some quality traits and grain yield. It may be concluded that by selecting suitable locations and different sowing managements for subjecting the crop to desirable environmental conditions (temperature and water) quality traits of wheat crop could be modified.

## Introduction

Drought and heat stress during grain growth developmental stages of crop are major factors that affects wheat yield and grain quality. Crop yield and grain quality will be having significant consequences due to changes in the frequency and severity of extreme climate events [1]. Increased annual change in temperature and rainfall are main components of climate change [2]. The impacts of climate variability are being seen in last few decades on agricultural crops. Agronomic and genetic adjustments are required to mitigate the direct and indirect effect of climate variability on crop metabolism to increase grain yield and quality [3]. According to Blumenthal et al. (1993) climate, soil and agronomic practices have a strong influence on expression of technological quality of different cultivars [4]. Thermal stress and water deficit during grain filling is responsible for fluctuation in grain yield as well as protein content and composition [5]. The thermal effect has been seen on the rheological properties of dough and on the quality of the end-product [6–7].

Wheat is the main cereals grains which provide proteins, energy, minerals and vitamins to most of the world population. Wheat grain is used for making flour which has number of uses (chapatti, bread, biscuit, noodles and pasta). Proteins and starch are mainly responsible for flour quality. The wheat grain proteins could be classified as monomeric (albumins, globulins and gliadins) and polymeric (glutenins) based upon their solubility [8]. The balance of these two proteins determines the rheological properties, viscosity and elasticity of flour [9]. Gliadins determine the viscosity while glutenins is generally associated with elasticity of dough. Another major constituent of flour is starch which plays an important role in food product quality and can be called as food determinant. It provides carbon during yeast fermentation which is helpful in setting of bread loaf and retrogradation during storage [10]. The texture and quality of the end use products like flour could be changed by having starches in them. The small starch granules increase the extensibility of the dough while large granules increased the resistance to extension [11]. Wheat grains are good sources of proteins, amino acids, carbohydrates, lipids and minerals [12]. These also clarify nutritional dietary value of wheat grain.

Wheat is widely grown crop under rainfed climate of semi-arid regions where amount and frequency of rainfall during grain filling stages vary which resulted to the change in the grain quality. According to Jiang et al. (2009) anthesis and grain filling period are critical growth stages where adverse environmental conditions leads to negative effect on grain quality [13]. However, effect could be minimized by selecting suitable locations, appropriate genotypes and modification in sowing time [14]. Sowing date affects grain quality mainly through its determination of the thermal conditions during the grain filling period, since late sown genotypes generally flowers late, thereby forcing the grain filling period to coincide with high temperature and water stress. These high temperature and water stress might lead to the significant effect on grain quality traits. According to Labuschagne et al. (2009) increased temperature and drought stress during grain filling period resulted to early maturity and shortened duration of glutenin synthesis, which in turn reduced dough strength [9]. The outcome of earlier researcher reported increased protein content in wheat grains under water stress [15–16].

Grain protein and gluten quality are the two most important parameters which are significantly affected by change in sowing time. The environmental effect on grain quality of wheat could be manipulated by an appropriate choice of sowing time and variable climatic locations. The sowing time, variable locations and photoperiod determines the flowering time which ultimately altered the environmental conditions prevailed before and during grain filling period. The sowing window in the Mediterranean environments like Pakistan extends over 3–4 months, sowing during first half of December results in variability in flowering time. The late sown materials comes under heat stress during flowering resulting to reduced grain size but increased protein accumulation compared to starches and vice versa [17].

Little work has been done to study impact of climate variability (rise in temperature and water stress) under rainfed conditions during grain filling stages of wheat crop. Wardlaw et al. (2002) studied effect of high temperature and drought stress on protein/starch accumulation and yield traits but without considering sowing time as main determinant factor [18]. Simialrly, no work has been done to study effect of variable climatic conditions on wheat grain quality. This paper, therefore, seeks to study the effect of sowing time and different variable climatic locations on grain quality of wheat under rainfed conditions of Pakistan. The innovation of this study will be design of suitable sowing dates in response to climate variability in order to bring sustainability in the wheat crop yield and improvement in the grain quality. The objective of this study were (i) to study the effect of sowing management under variable climatic locations on physiological, grain quality traits and grain yield; (ii) to develop a regression model between physiological, grain quality traits, grain yield and treatments and (iii) to give optimum sowing date as management option for quality and yield maintenance under varying climate.

## Material and Methods

### Study Sites

Three study sites, Islamabad, Chakwal, and Talagang, with contrasting temperature and rainfall characteristics were chosen for this study. The field studies did not involve any endangered or protected species and no specific permissions were required for these locations/activities. Islamabad (33°40’N, 73°10’E, 508 m a.s.l.) has a dry sub-humid climate and is located in the high rainfall agro-ecological zone [19]. The mean annual reference evapotranspiration (ET_0_, FAO Penman-Monteith) [20] at Islamabad is about 1588 mm. The semi-arid site Chakwal (32°56’N, 72°52’E, 513 m a.s.l.) is situated in the medium rainfall zone, has an annual ET_0_ of about 1585 mm. Talagang (32°55’N, 72°25’E, 458 m a.s.l.) has an arid climate and is located in the low rainfall zone [21]. The annual ET_0_ at Talagang is about 1592 mm.

### Soil of the study sites

**Fig 1:**
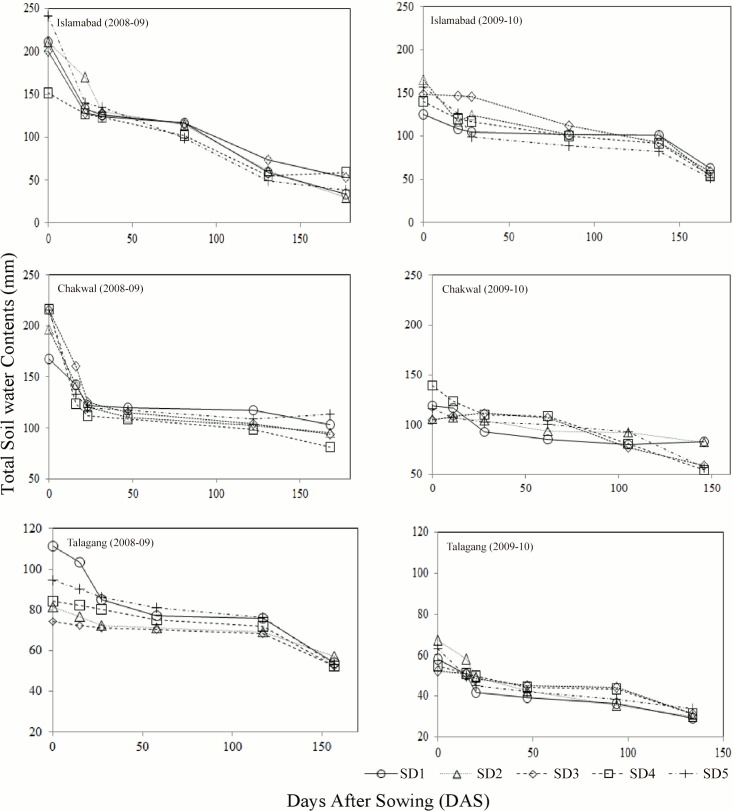
Total Soil Water Contents (mm) from sowing to maturity at three locations during two environments and among five sowing dates (SD).

The soil classification of study site Islamabad was Rajar, with great groups Ustorthents and soil order Entisol, while soil series of Chakwal site described as Dhumman with great groups Haplustalfs and soil order Alfisol, and Talagang soil series characterize as Talagang with subgroups Ustochrepts and order Inceptisols USDA. Total soil water contents (mm) were recorded from sowing to maturity at three locations during two years and among five sowing dates (SD) to show the variability in the soil water contents (Fig 1). The physiochemical properties at Islamabad (pH = 7.4, EC (dSm^-1^) = 0.23, N (%) = 0.039, Texture = loam, bulk density(gcm^-3^) = 1.22, soil lower limit (mmmm^-1^) = 0.07, drain upper limit(mmmm^-1^) = 0.34 and saturated soil water (mmmm^-1^) = 0.46), at Chakwal (pH = 7.9, EC(dSm^-1^) = 0.28, N (%) = 0.03, Texture = sandy clay loam, bulk density (g cm^-3^) = 1.29, soil lower limit (mmmm^-1^) = 0.057, drain upper limit(mmmm^-1^) = 0.24 and saturated soil water (mmmm^-1^) = 0.46) and at Talagang were (pH = 8.1, EC(dSm^-1^) = 0.20, N (%) = 0.03, Texture = sandy loam, bulk density(gcm^-3^) = 1.35, soil lower limit (mmmm^-1^) = 0.050, drain upper limit(mmmm^-1^) = 0.15 and saturated soil water (mmmm^-1^) = 0.41).

### Plant material and sowing times

Three wheat (*Triticum aestivum* L.) genotypes *viz*; Chakwal-50, Wafaq-2001 and GA-2002 were planted in a randomized complete block design replicated four times under five varying sowing dates (Early sown = SD1, (sowing between 15–20 October), SD2 (sowing between 25–30 October), SD3 (sowing between 5–10 November), Optimum sowing time (SD4) = sowing between 15–20 November) and Late sown = SD5 (sowing between 01–10 December). The plot size was 4.5 x 10 m with the row to row distance of 30 cm. The seed rate used was 123.5 kg per hectare.

### Crop management

Seed drill was used for sowing under different sowing times at three variable locations. Weeds were controlled manually. Fallow land was selected at three locations during two years (2008–09 and 2009–10). The land was prepared using cultivator followed by mouldboard plough. Urea (46%) at 100 kg ha^-1^ was used in each plot as source of N applied at the time of sowing. P fertilizer at 75 kg ha^-1^ was provided in the form of DAP. K was not applied as adequate soil- available K was in soil at three sites.

### Measurements

SPAD 502 Minolta chlorophyll meter [22] was used to determine chlorophyll contents at anthesis stage (Zadok’s growth stage, Z-60) of wheat from all treatments (Years (2008–09 and 2009–10), locations, sowing dates and genotypes). Leaf samples from all treatments were taken at random at anthesis stage. The samples from each treatment were homogenised in ten ml Sulphosalicyclic acid (C_7_H_6_O_6_S, 3% w/v) solution at room temperature and stored at 4°C for overnight. The supernatant obtained was added with glacial acetic acid and acid ninhydrin and reaction was conducted in a test tube for one hour at 100°C while reaction was terminated by putting test tube in ice bath. The four ml of benzene derivative toluene was used to extract the sample in a test tube stirrer for 15–20 seconds. The extracted samples were analysed using UV-visible spectrophotometer at 520 nm (Thermo Electron, Model Bio Mate 3, Massachusetts, USA). The concentration of proline was determined by calibration curve and expressed as mg proline per gram fresh weight.

Thirty spikes from each plot of all treatments were taken at physiological maturity of crops. Zadok’s scale was used to determine physiological maturity and it’s was Z90. The seeds were threshed manually and obtained seeds were used for quality analysis of wheat. Furthermore all plots were harvested to have grain yield in kg ha^-1^_._ Cyclotec 1093 sample mill (Foss, Tecator, Sweden) was used to converts grains obtained from all treatments into flour. Kjeldahl analysis using a Kjeltec 2100 (Sweden) distillation unit was used to measure N contents which were converted to total protein after multiplying with 5.72. The AACC Method 38–12 was used to determine wet and dry gluten. The basic SDS (sodium dodecyl sulfate) sedimentation test AACC Method 56–70 was used to determine SDS value. The rate of sedimentation of a meal suspension in SDS medium depends upon gluten quality as both are directly related. A sub sample 3.5 g of crushed whole grain was used to determine ash contents. The sample was placed in ashing dish that was ignited, cooled in desiccator and weighed at room temperature. The sample was placed in muffle furnace at 550°C until light gray ash was obtained. The sample was cooled to room temperature and weighed again. The percentage ash was calculated using following formula [23–24]

$$\%\;\text{Ash}=\frac{Weight\;of\;residue}{Sample\;weight}\;X\;100$$ (1)

### Statistical analyses

The data obtained from each parameter was subjected to analysis of variance (ANOVA) using years—combined randomized complete block design. The R [25] package was used for this purpose. The multivariate regression model was developed to study relationship between treatments and all parameters using validation skill scores R^2^.

## Results

### Climatic information

**Fig 2:**
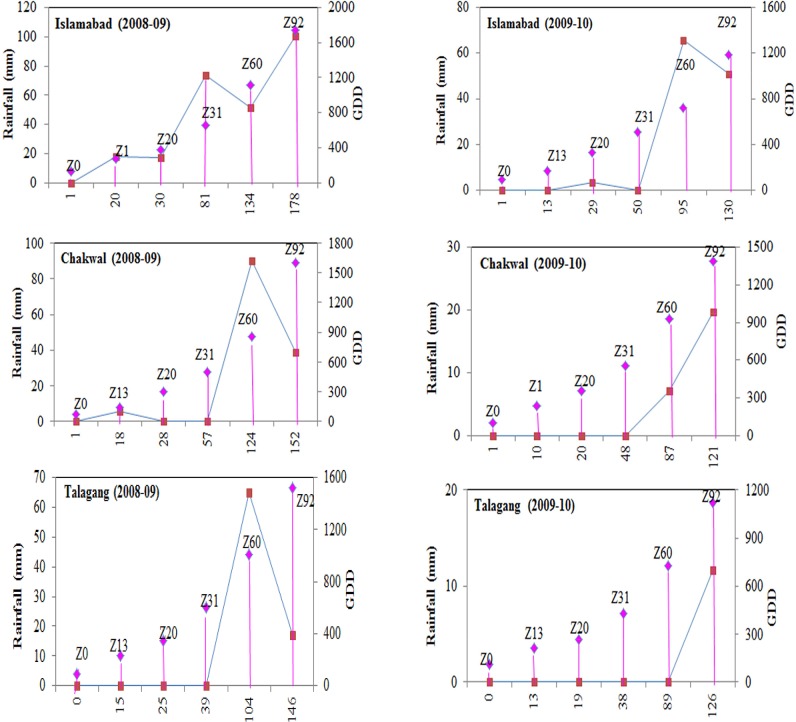
Mean phenological development (Zadok (Z) Scale) of wheat genotypes in relationship to rainfall (Maroon marker with solid blue line)() and Growing Degree Days (GDD) (Pink marker with blue border line) at three study sites during two environments (2008–09 and 2009–10) averaged over five sowing dates (SD).

The different sowing date selected in this study resulted in exposure of wheat plants to varied temperature and rainfall before and during grain filling growth stages at three variable climatic conditions study sites. The mean maximum temperatures at Islamabad during grain filling periods under early sown, timely sown and late sown conditions were 25.4, 27.3 and 29.4°C as an average of both years and rainfall received were 97, 85 and 79 mm respectively. Similarly mean minimum temperatures were 9.8, 12.3 and 16.5°C in early sown, timely sown and late sown conditions respectively. However, at Chakwal the mean maximum temperature under early, timely and late sowing dates were 26.1, 27.8 and 30.1°C while mean minimum temperatures remained 10.2, 13.5 and 17.5°C respectively. The rainfall received during grain filling period at Chakwal in early, timely and late sown conditions were 85, 65 and 55 whereas it remained significantly low during second year (Fig 2). The mean maximum temperature at Talgang remained 27.9, 29.2 and 31.3°C respectively in early, timely and late sown conditions while mean minimum temperature remained 11.3, 13.9 and 18.3°C respectively. The rainfall received during grain filling period at Talagang in early, timely and late sown conditions were 46, 35 and 27 mm. Variable temperature range and rainfall amount at different study sites in different sowing dates resulted in significant difference in SPAD chlorophyll contents, proline, grain quality and yield. Significant change in the study traits were also because of change in the Growing Degree Days (GDD) as they remained significantly different for the phenological development (Zadok (Z) Scale) of wheat genotypes at three study sites during two years (2008–09 and 2009–10) averaged over five sowing dates (SD). The results revealed that rainfall remained higher at Islamabad during grain filling period compared to all other locations (Fig 2).

### SPAD chlorophyll Contents

**Fig 3:**
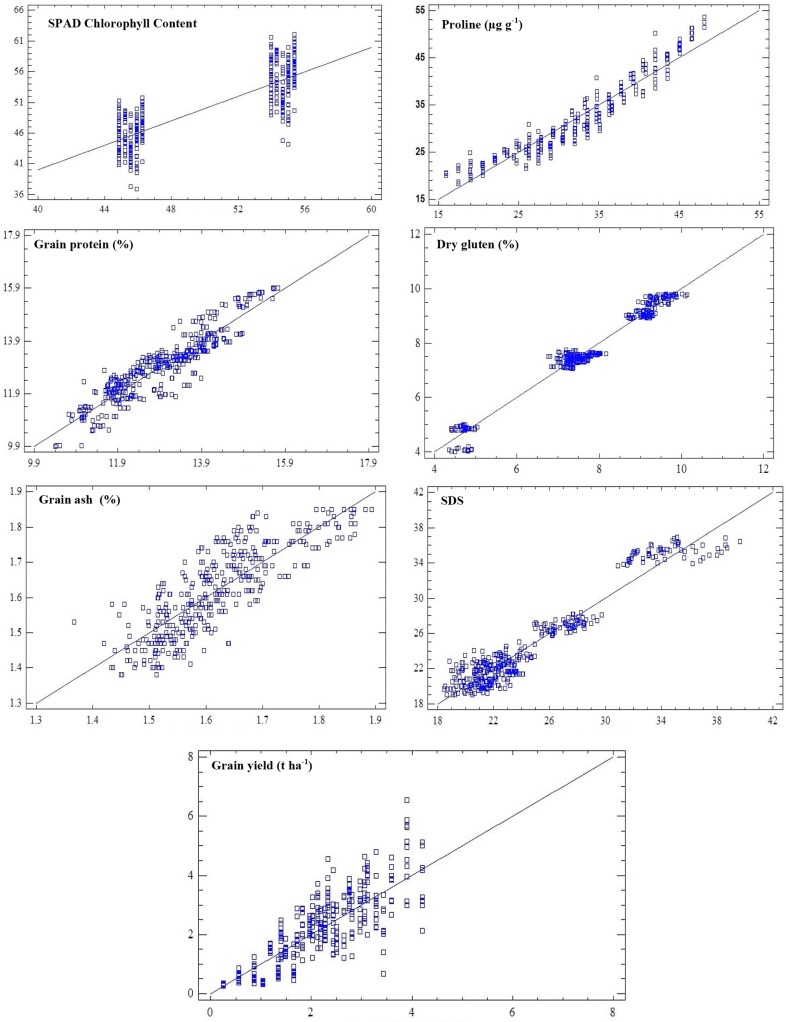
1:1 relationship among simulated (x-axis) and observed (y-axis) physiological, grain quality traits and grain yield in response to treatments.

The mean squares values from analysis of variance table for SPAD chlorophyll contents depicted that years (Y), location (L) and management have significant effect on SPAD value while genotype effect remained non-significant. The interactive effects of YxL, YxSD, LxSD and YxLxSD showed significant effects on SPAD chlorophyll contents (Table 1). The year, 2008–09 (54.67), location, Islamabad (51.80) and management, SD1 (52.54) produced the highest SPAD chlorophyll contents (Table 2). Furthermore, modeled SPAD-502 chlorophyll contents in relation to different parameters like year (2008–09 and 2009–10), genotypes, locations (Islamabad, Chakwal and Talagang) and management options (Sowing dates) depicted significantly decreasing relationship. The modeled equation showed that SPAD chlorophyll contents decreased when moved from 2008–09 to 2009–10. Similarly, among genotypes the SPAD Chlorphyll contents decreased significantly and similar trend was observed for location and sowing dates. The R^2^ value for modeled equation was 0.76 depicting 76% relationship between SPAD chlorophyll contents and independent parameters like year, locations, genotypes and sowing dates. Meanwhile to check relationship between different quality parameters (dry gluten, Grain ash, grain protein and SDS), grain yield and proline contents with SPAD chlorophyll contents, regression model was developed with R^2^ of 89% (Table 3). The modeled equation depicted that SPAD chlorophyll contents have negative relationship with all parameters except dry gluten and grain yield combined over years, locations, sowing dates and genotypes. The modeled relationship of SPAD chlorophyll contents with other parameters and treatments was showed by using 1:1 graph which indicated close association between observed and simulated values (Fig 3).

**Table 1:** Summary of mean squares of quality traits and grain yield.

SOV	DF	SPAD Chlorophyll Content	Proline	Grain Protein	Dry Gluten	Grain ash	SDS	Grain Yield
**Years (Y)**	1	98.6169**	6468.54**	95.0181**	98.6169**	0.962**	4818.98**	119840592.4**
**Location (L)**	2	346.144**	287.878**	140.414**	346.144**	1.79**	1078.42**	84572494.85**
YxL	2	0.79897**	6389.17**	29.5666**	0.79897**	0.142**	57.4125**	2392294.51**
**Management (SD)**	4	0.21132**	589.699**	29.3899**	0.21132**	0.094**	13.0082**	25927895.82**
YxSD	2	53.9827**	5.05117**	9.52338**	53.9827**	0.081**	1349.71**	4046924.37**
YxSD	2	0.22336**	523.518**	0.04719**	0.22336**	0.007**	1.34373**	ns
YxLxSD	4	0.0599**	10.345**	0.00799**	0.0599**	0.007**	0.78379**	743652.69**
**Genotype (G)**	4	ns	4.98176**	0.07022**	0.00991*	0.08**	ns	268294.65*
YxG	8	ns	3.34533**	0.01083**	ns	ns	ns	1358625.51**
LxG	8	ns	10.2272**	0.01443**	ns	ns	ns	678968.45**
ExLxG	4	ns	ns	0.00528**	ns	ns	ns	ns
SDxG	8	ns	ns	0.000759**	ns	ns	ns	1729381.43**
YxSDxG	8	ns	0.8369*	ns	ns	ns	ns	1114275.53**
LxSDxG	16	ns	ns	ns	ns	ns	ns	ns
YxLxSDxG	16	ns	ns	ns	ns	ns	ns	ns

Note: * and ** are significant at 0.05 and 0.01 probability levels, respectively; ns, non-significant; DF, degree of freedom.

**Table 2:** Mean comparison of different quality traits and grain yield at different levels of treatments.

Treatments	SPAD Chlorophyll Content	Proline (μg g^-1^)	Grain Protein (%)	Dry Gluten (%)	Grain ash (%)	SDS	Grain Yield (t ha^-1^)
**Years (Y)**							
Y1 (2008–09)	54.67^a^	27.77^b^	12.44^b^	8.12^a^	1.56^b^	21.03^b^	2.81^a^
Y2 (2009–10)	45.56^b^	36.25^a^	13.47^a^	7.07^b^	1.67^a^	28.34^a^	1.65^b^
**Locations (L)**							
Islamabad	51.80^a^	24.55^c^	12.40^c^	7.67^a^	1.58^c^	24.01^c^	3.19^a^
Chakwal	49.43^b^	32.36^b^	13.14^b^	7.61^b^	1.61^b^	24.65^b^	1.89^b^
Talgang	42.12^c^	39.13^a^	13.34^a^	7.51^c^	1.65^a^	25.40^a^	1.62^c^
**Genotypes (G)**							
Chakwal-50	50.49^ns^	33.61^a^	12.75^b^	5.94^c^	1.52^c^	27.24^a^	3.28a
Wafaq-2001	49.92	31.90^b^	11.99^c^	7.52^b^	1.75^a^	21.73^c^	2.06c
GA-2002	49.93	30.52^c^	14.13^a^	9.33^a^	1.57^b^	24.51^b^	2.25b
**Sowing Dates (SD)**							
SD1	52.54^a^	30.74^d^	12.90^d^	7.61^b^	1.60^d^	24.37^d^	2.52^b^
SD2	49.63^b^	27.97^e^	11.88^e^	7.68^a^	1.57^e^	24.13^e^	3.12^a^
SD3	47.60^b^	32.10^c^	13.16^c^	7.60^b^	1.62^c^	24.82^c^	2.05^c^
SD4	50.32^a^	33.82^b^	13.36^b^	7.57^bc^	1.66^a^	24.97^b^	1.84^d^
SD5	50.48^a^	35.42^a^	13.48^a^	7.54^c^	1.64^b^	25.15^a^	1.62^e^

Notes: Means with similar letter(s) have no significant difference while different letters a,b,c,d,e showed that means are significantly different from each other at 0.05 probability levels, ns = non-significant.

**Table 3:** Multivariate regression fitted model to study relationship between physiological, grain quality traits, yield of spring wheat and treatments.

			Analysis of Variance				
**Parameters**	**Fitted model**	**R** ^**2**^	***SOS***	***Df***	***MS***	***F-Ratio***	***P-Value***
**SPAD**	SPAD = 65.7067–9.11189*Y—0.283708*G—0.159792*L—0.345292*SD	76	7583.68	4	1895.92	171.68	**
** **	SPAD = 119.132–0.176165*DG—21.4667*GA—0.982818*GP—1.40697*GY—0.0660608*PL—0.605003*SDS	89	5585.52	6	930.919	55.52	**
**Proline**	PL = 3.24983 + 8.47778*Y—1.54604*G + 7.29046*L + 1.51883*SD	91	21459.3	4	5364.83	867.59	**
** **	PL = -1.15692–2.91248*DG + 15.4017*GA + 3.65427*GP—2.31578*GY—0.35528*SDS—0.0606331*SPAD	87	18222.2	6	3037.04	197.35	**
**Grain Protein**	GP = 8.29847 + 1.0275*Y + 0.691375*G + 0.47075*L + 0.264611*SD	55	313.337	4	78.3342	107.41	**
** **	GP = 9.96677 + 0.643904*DG—3.39538*GA—0.304698*GY + 0.0646317*PL + 0.121606*SDS—0.0159545*SPAD	83	476.168	6	79.3613	291.58	**
**Dry Gluten**	DG = 6.01536–1.04678*Y + 1.69704*G—0.0810833*L—0.0247222*SD	87	791.823	4	197.956	598.52	**
** **	DG = -3.45383 + 2.93127*GA + 1.0661*GP + 0.188516*GY—0.0852876*PL—0.200735*SDS—0.00473487*SPAD	83	750.159	6	125.026	277.44	**
**Grain ash**	GA = 1.27967 + 0.103389*Y + 0.0283333*G + 0.0344167*L + 0.0195556*SD	32	1.71432	4	0.42858	40.97	**
** **	GA = 2.67121 + 0.0423568*DG—0.0812334*GP—0.0564484*GY + 0.00651719*PL + 0.000539882*SDS—0.00833723*SPAD	56	3.12882	6	0.52147	80.08	**
**SDS**	SDS = 14.7226 + 7.31739*Y—1.55571*G + 0.690875*L + 0.239833*SD	56	5555.8	4	1388.95	113.99	**
** **	SDS = 24.5835–2.77647*DG + 0.516773*GA + 2.78484*GP + 0.0764933*GY—0.143901*PL—0.224914*SPAD	78	7681.05	6	1280.17	205.38	**
**Grain Yield**	GY = 6.49562–1.15393*Y—0.0171375*G—0.784942*L—0.310326*SD	64	337.121	4	84.2802	155.19	**
** **	GY = 15.9924–0.0338893*SPAD + 0.00495613*SDS + 0.168942*DG—3.50084*GA—0.452101*GP—0.0607727*PL	74	387.355	6	64.5592	159.86	**

Note: * and ** are significant at 0.05 and 0.01 probability levels, respectively; ns = non-significant; SOS = sum of square; MS = mean square Df = degree of freedom

Y = Years; G = Genotypes = Locations; SD = Sowing dates; PL = Proline; DG = dry gluten; GA = Grain ash; GP = Grain protein; GY = Grain yield

### Proline (μg g^-1^)

Analysis of variance table for proline contents depicted significant effect of treatments (Table 1). The interactive effect remained highly significant except the interactions of YxLxG, SDxG, LxSDxG and YxLxSDxG. Mean table for proline contents depicted the highest proline contents recorded during 2009–10 (36.25 μg g^-1^), at Talagang (39.13 μg g^-1^) for genotypes Chakwal-50 (33.61 μg g^-1^) and management, SD5 (35.42 μg g^-1^) (Table 2). The regression modeled equation showed that proline contents increased significantly on moving from one year (2008–09) to second year (2009–10). Similar increasing trend was recorded for locations and managements (SD). The R^2^ value for modeled equation was 0.91 depicting 91% relationship between proline contents and independent parameters like year, locations, genotypes and sowing dates. The relationship between studied traits was further modeled by regression model and equation depicted that different parameters have relationship among each other with R^2^ of 87% ((Table 3). The modeled equation depicted that proline contents have positive increasing trend with grain ash (GA), grain protein (GP) and negative decreasing trend with dry gluten (DG), sodium dodecyl sulphate (SDS) and SPAD value. The modeled relationship of proline contents with other parameters and treatments was showed by using 1:1 graph which indicated close association between observed and simulated values (Fig 3)

### Grain Protein (%)

Grain protein contents depicted significantly strong relationship with treatments. Analysis of variance table revealed that grain protein was significantly affected due to years, locations, managements and genotypes (Table 1). The mean table showed the highest protein contents noted during 2009–10 year (13.47%). However, among locations the maximum protein contents were observed for Talagang (13.34%) followed by Chakwal (13.14%) and Islamabad (12.40%). The management as sowing date depicted significant effect on grain protein. The highest grain protein was observed for late sowing date i.e. SD5 (13.48%) followed by SD4 (13.36%). The lowest protein content was observed for SD2 (11.88%). The mean value of protein contents for genotypes revealed that genotype GA-2002 (14.13%) have maximum value followed by Chakwal-50 (12.75%) and Wafaq-2001 (11.99%) (Table 2). The regression modeled between grain protein (GP) and independent variables (Years, genotypes, locations and sowing dates) showed that change in years increased grain protein, the coefficient was 1.0275, whereas increase due to genotypes, locations and sowing date was 0.691375, 047075 and 0.264611, respectively (Table 3). However, relationship between different studied traits regression model depicted that grain protein increased with the increase of dry gluten, proline, SDS while it decreased significantly with increased value of grain ash, grain yield and SPAD chlorophyll contents. The R^2^ value of 83% confirmed that model equation could be used to predict protein contents in response to different studied traits (Table 3 and Fig 3).

### Dry Gluten (%)

Analysis of variance for dry gluten depicted significant effect of treatments. However, among interactive effects only YxL, YxSD, LxSD and YxLxSD remained significant (Table 1). The mean value table showed that dry gluten was highest during 2008–09 (8.12%) compared to second year (Table 2). Among locations highest dry gluten recorded for Islamabad (7.67%) followed by Chakwal (7.61%) and Talagang (7.51%). Similarly, among managements, maximum dry gluten was observed for SD2 (7.68%) followed by SD3 (7.60%) which was at par with SD1 and SD4. The regression model equation developed between dry gluten and independent variables depicted strong relationship with R^2^ of 87%. The model showed that with the change in years, locations and sowing dates dry gluten decreased significantly while increased trend was observed for genotypes. Therefore, our model could be used to predict dry gluten. The regression equation with different studied traits depicted significant positive and negative coefficients values with R^2^ of 83% (Table 3). Meanwhile, 1:1 graph showed strong association between observed and simulated values (Fig 3).

### Grain ash (%)

The amount of grain ash was significantly affected by different treatments (Table 1). The interactive effect of YxL, YxSD, LxSD and YxLxSD was significant for grain ash while all other interactions remained non-significant. The regression model for grain ash depicted significant increase with the change in the years, genotypes, locations and sowing dates. Similarly, model equation to study relationship among different study traits revealed that grain ash contents increased with the increase of dry gluten, SDS and proline. However, the value of grain ash decreased significantly if grain protein, grain yield and SPAD chlorophyll contents increased (Table 3).

### Sodium dodecyl sulfate (SDS)

Maximum SDS varied among years, locations, genotypes and management with the highest average value for SD5 (25.15) compared to other sowing time. Among locations the maximum value of SDS recorded for Talagang (25.40) followed by Chakwal (24.65) and Islamabad (25.40). The regression model for SDS in response to treatments depicted significant positive trend with years, locations and sowing dates while trend was negative with genotypes. However, relationship between different study traits modeled using regression modeling depicted significant relationship between study traits (Table 3).

### Grain Yield (t ha^-1^)

**Fig 4:**
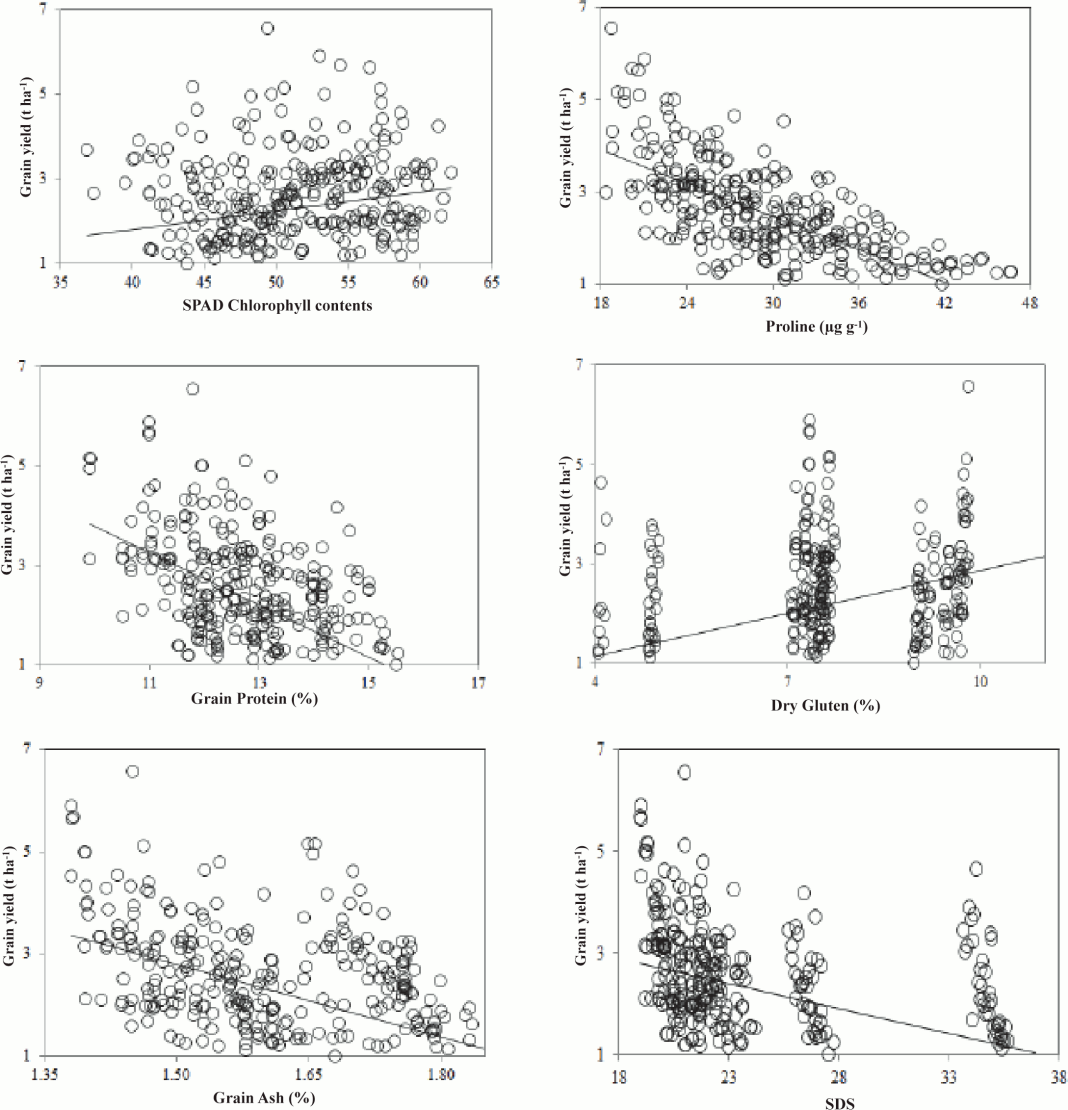
Relationship of physiological, grain quality traits with grain yield of wheat combined over all treatments.

Grain yield was significantly related to all treatments (Table 1). The mean table revealed higher grain yield (2.81 t ha^-1^) recorded during 2008–09 compared to 2009–10 (Table 2). The grain yield under different locations showed that it remained highest at Islamabad (3.19 t ha^-1^) followed by Chakwal (1.89 t ha^-1^) and Talagang (1.62 t ha^-1^). Management as sowing time depicted that grain yield remained highest for SD2 (3.12 t ha^-1^) followed by SD1 (2.52 t ha^-1^) while lowest value recorded for late sowing i.e. SD5 (1.62 t ha^-1^). The regression model equation revealed negative trends of grain yield with all treatments. The model results showed reduction of grain yield if moved from early sowing to late sowing, similar trend was observed for locations (Table 3). The association among different parameters was evaluated using 1:1 graph which depicted close relationship between observed and simulated values (Fig 3). The relationship of physiology and quality traits with grain yield revealed that SPAD Chlorophyll contents and dry gluten have positive association with grain yield while all other studied parameters have inverse relationship (Fig 4).

## Discussion

The highest SPAD chlorophyll contents during 2008–09 would have been due to favourable environmental conditions during crop life cycle. Among locations maximum value at Islamabad compared to other locations was probably due to suitable temperature and rainfall during critical growth period of crop (Figs 1 and 2). Different sowing date generated a large effect on the amount of SPAD chlorophyll contents that might be due to favourable thermal conditions. This effect was more evident on comparing early and late sowings. The regression modelling approach also depicted that shift of crop from early sowing to late, favourable environment to stress and from suitable locations to stress, SPAD chlorophyll contents decreased significantly. Results of present study are consistent with earlier findings who concluded variable SPAD values with favourable climatic conditions and stress [26]. The modelled positive relationship of SPAD chlorophyll contents with grain yield and dry gluten depicted that photosynthetic machinery has strong relationship with grain yield [27–29]. However, contrary to our finding, Hamblin et al., (2014) reported reduced chlorophyll contents as advantageous to higher yields because it might reduce heat load and water requirements to cool leaves [30]. The negative association of SPAD chlorophyll contents with other parameters (proline, grain protein and SDS) showed that under stress SPAD chlorophyll contents decreased while antagonistic components increased to compensate the stress effects [31]. Akhkha et al., (2011) and Vendruscolo et al. (2007) reported reduced chlorophyll but increased proline contents under water limited years [32–33]. Miller et al. (1999) found significant relationship between grain protein and SPAD units and concluded that protein and SPAD contents have inverse relationship under water stress [34].

Plants are adapted to water stress by osmoregulation for maintaining continuous supply of water to plants under decreased water potential and prevent dehydration [35]. Proline plays main role in osmoregulation and its contents increased tenfold under drought as in the present study [36–37]. The study showed that under stress proline contents increased significantly, having synergistic relationship with grain ash (GA), grain protein (GP) [38] and antagonistic trend with dry gluten (DG), sodium dodecyl sulphate (SDS) and SPAD.

Wheat is an essential part of the diet of the world population therefore its quality traits are most critical. The important index to evaluate quality of wheat is grain protein [38]. The increased GP recorded under stress compared to optimum conditions proved that grain responded directly to high temperature and water stress by modifying source-sink balance [14]. Changing the sowing time depicted large effect on the grain protein which might be due to the modifications of thermal conditions during grain filling and variability in rainfall [39–40] (Fig 1). The relationship of GP with other studied components showed that GP increased with increased dry gluten, proline and SDS. However, GP have negative association with grain ash, grain yield and SPAD chlorophyll contents [41]. Since, GP contents increases under high temperature and water stress during grain filling period therefore, it showed inverse relationship with grain ash, grain yield and SPAD chlorophyll contents [39]. Wheat is unique as a source of gluten protein and quality traits like dry gluten, grain ash and SDS showed significant variability in response to different treatments which was due to variability in the temperature and rainfall during grain filling periods [42–43]. Similarly, quality traits can be affected by variable locations, years and sowing management [44].

Modification in the grain quality traits and yield under “too good” conditions (during 2008–09, at Islamabad and early sowing) compared to “too bad” conditions (during 2009–10, at Talagang and late sowing) depicted that crop grain quality traits and yield was strongly influenced by years, locations and sowing managements [45]. Gil et al., (2011) reported decreased wheat grain yield with delayed sowing but increased grain protein [46]. The effects of extreme variable climatic conditions on wheat grain yield and quality trait were reported by earlier researcher [7]. They concluded that drought and heat stress resulted to reduce grain yield but enhanced quality traits like protein. Therefore, it’s essential to select optimum sowing management which is SD2 and genotypes i.e. Chakwal-50 to minimize extreme effect of climate variability on crop productivity and quality. The alteration in the grain quality of bread wheat in response to increased climatic variability was earlier reported [47]. They concluded strong correlation between grain yield and protein. Similarly, Moldestad et al., (2014) concluded a significant effect of temperature on quality traits of wheat [48]. The demand for high quality end products resulted to increased preference to quality traits in addition with yield. Our results depicted that grain quality is directly affected by agronomic (Sowing date) and environmental factors [49]. Negative impact of abiotic environmental stresses on crop productivity and quality in present findings confirms the importance of adjustment of sowing date and cultivars selection [50].

## Conclusions

Delayed sowing resulted to the reduction in the grain yield therefore, early sowing should be recommended to the framers to avoid water and temperature stress during grain filling stages of the wheat crop. However, effects of extreme climatic conditions (water stress and high temperature) are beneficial for quality traits like proline, grain ash (GA) and grain protein (GP) but on the expense of grain yield. Our study suggested that in order to bring sustainability in the crop yield with consistent quality traits genetic modification approach has to be opted. However, quality traits of the crop could be modified by subjecting crop to different sowing times, variable climatic locations and management into locations interactions. Since sowing date is the main determinant factor for crop quality and yield therefore, it should be recommended according to prevailing weather conditions using long-term weather forecasting data.
